# Crickets in Context: How Environment and Morph Relate to Locomotory Behavior

**DOI:** 10.1002/ece3.72804

**Published:** 2025-12-26

**Authors:** Tessa M. Appel, Sterling Kerr, Robin M. Tinghitella, Spencer Ingley, Gabe Meyer, E. Dale Broder

**Affiliations:** ^1^ Department of Biology University of Denver Denver Colorado USA; ^2^ Faculty of Sciences Brigham Young University–Hawaii Lā'ie Hawaii USA; ^3^ Department of Biology American University Washington DC USA

**Keywords:** context‐dependence, cricket, lab effects, locomotory behavior, plasticity

## Abstract

One challenge faced by animal behavior researchers is that behavior observed and studied in captivity may not reflect behavior in nature, but rarely are both contexts assessed. Pacific field crickets encounter an acoustically hunting lethal parasitoid fly in Hawaii, which has facilitated the rise of novel male morphs, like “purring,” that sing attenuated songs and are protected from the fly. Yet these novel morphs are still able to reproduce. It is possible that the quieter purring males use an alternative mating tactic like increased locomotory behavior to facilitate encountering mates. Using a population that contains purring males and typical ancestral males, we asked if the two types differ in their locomotory behavior in the wild, whether this result is maintained in the lab, and whether behavior depends on context (varying the test substrate: natural grass or artificial). In the wild, the two male morphs did not differ in the time spent walking on the grass, but ancestral males spent more time under the grass. In the lab, the two morphs spent the same amount of time moving but traveled significantly further and faster on the artificial substrate than on the grass. Unlike in the field, morphs did not differ in movement under the grass in the lab, which is interesting as crickets are subject to parasitism in the field and may be protected under the grass. Our findings highlight the value of pairing lab experiments with field observations to fully understand animal behavior.

## Graphical abstract

Because behavior is unlikely to be identical in the wild compared to the lab, we paired a lab experiment with the first in situ observation of movement behavior in the Pacific field cricket. We documented differences between our field and lab studies, namely morph differences in the field that did not appear in the lab, as well as contextual plasticity in the lab.

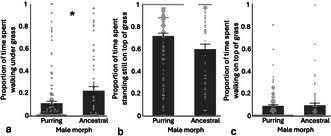

## 1 Introduction

Coupling field observations with controlled laboratory studies can be a powerful approach to comprehensively understand animal behavior since lab‐based studies allow one to control for the many sources of variation that generate differences in animal behavior in nature (e.g., Fisher et al. 2015; Thornhill and Alcock 1983). For instance, phenotypic plasticity, the ability of a single genotype to express more than one phenotype, is ubiquitous (Baldwin 1896; West‐Eberhard 1989), and behavior is particularly plastic (Chenard and Duckworth 2021). Both the rearing environment (developmental plasticity) and the immediate environment in which behavior is observed (contextual plasticity) could account for differences between laboratory and field observations (Snell‐Rood 2013). For instance, lab environments can affect endocrine function, including stress responses, brain morphology and function, circadian rhythms, and immune function; for example, male kestrels in the breeding season have higher androgens in the wild but lower androgens in captivity, which affects the behavioral phenotype (Calisi and Bentley 2009). Additionally, understanding the potential role of plasticity is especially important when species exhibit polymorphisms in key behaviors such as alternative mating tactics (Gross 1996)—these alternative paths to fitness may be differently affected by artificial lab environments. Here, we compare the locomotory behavior of two reproductive morphs of crickets in the wild and in two different contexts in the lab to gain a more comprehensive understanding of locomotory behavior.

Locomotory behavior, or searching behavior, is critical for many animals since it is the means by which they locate mates, food, oviposition/nesting sites, and refugia (Bell 1990). This behavior is especially important to consider in species with alternative mating tactics since they often differ in locomotory behavior. For instance, two common alternative mating tactics are variations on the theme of individuals who do active and conspicuous courtship versus individuals who search for mates or sneak matings (going undetected by courters; e.g., Gross 1996); searchers are more locomotory than courters, making locomotory behavior critical to fitness. Yet, observing locomotory behavior in the wild is challenging, particularly for species that are difficult to detect on camera (e.g., small or nocturnal), so locomotory behavior in the wild is better studied for megafauna (review of mammals in captivity vs. wild, Kelly et al. 2025; Strandburg‐Peshkin et al. 2015). Observations of locomotory behavior can be done in real time (e.g., Joern et al. 1986) or passively using video (e.g., Makai et al. 2020) and may employ methods like individual focal follows or scan sampling (Altmann 1974). Indeed, much can be gleaned from estimating time budgets in nature (the time that animals spend on various behaviors like movement) as all behaviors trade off with others, and this can have ecological and evolutionary impacts (Sih et al. 2012).

The Pacific field cricket, *Teleogryllus oceanicus*, is an insect that is often studied in the lab and also recently evolved new male morphs that may use alternative mating tactics. Male *T. oceanicus* primarily use a loud, tonal calling song to attract female mates under the cover of darkness, but in their introduced range in Hawaii, their songs also attract the deadly parasitoid fly, *Ormia ochracea* (Zuk et al. 1993). In response to this novel selection pressure, new morphs of *T. oceanicus* have evolved that produce different calling songs that protect them from flies. One new male morph, called purring, produces a song that is much quieter than the ancestral song and thus protects them from the fly but is much less effective at attracting mates (Tinghitella et al. 2018, 2021). There are several locations in Hawaiʻi where purring males inhabit the same fields as ancestral males (Gallagher et al. 2023). In locations where multiple morphs coexist, each morph is subject to a different balance of sexual selection (attracting mates) and natural selection (avoiding deadly fly parasitism; Gallagher et al. 2024). It is unknown whether ancestral and purring males differ in locomotory behavior, but existing evidence suggests purring males may need to search more for mates, as their song is only detectable by females at short distances (Welsh et al. 2025). It was recently shown that silent males (another recently evolved morph that produces no song) locomote more than ancestral males (Sturiale and Bailey 2023; Schneider et al. 2024), which presumably contributes to their success in mating, despite not producing a sexual signal. It is possible that purring males also differ from ancestral males in their locomotory behavior. Differences in locomotory behavior between morphs could stem from genetic or environmental influences. For instance, Sturiale and Bailey (2023) report that there are genetic differences in juvenile locomotory behavior between flatwing/silent and ancestral *T. oceanicus* lines. Additionally, *T. oceanicus* locomotory behavior is developmentally plastic in response to the juvenile acoustic environment in some studies (Balenger and Zuk 2015; Moschilla et al. 2022), but not others (Heinen‐Kay et al. 2018). It is unclear whether the behavior is contextually plastic (rapidly reversible in response to a given situation; Snell‐Rood 2013). Regardless of underlying mechanisms, increased movement should increase the chance that males with attenuated signals encounter mates (Hissmann 1990). Relatedly, increased movement coupled with phonotactic behavior towards ancestral calling males could be part of an alternative mating tactic called satellite behavior (Zuk et al. 2001; Bailey et al. 2010; Balenger and Zuk 2015).

Because there are strengths and weaknesses to both field and lab studies, it is important to observe behavior in both contexts and avoid relying on lab‐based behavior as representative of behavior in the wild (Thornhill and Alcock 1983; Fisher et al. 2015). We conducted the first in situ observation of movement behavior in the Pacific field cricket and paired that field experiment with a lab experiment. One distinguishing feature of this study was that the field experiment was conducted as part of an inter‐institutional CURE (Course‐based Undergraduate Research Experience; following Smith et al. 2021), a high‐impact teaching approach that increases access to authentic research experiences (Box 1). We first asked if ancestral and purring males differ in their locomotory behavior in the wild. We predicted that ancestral males, which are more attractive to deadly eavesdroppers (e.g., Tinghitella et al. 2021; Gallagher et al. 2023; Wikle et al. 2025), would spend more time under the grass (rather than on the surface) than the more protected purring males. We also expected purring males to move more than ancestral males since silent males move more than ancestral males (Sturiale and Bailey 2023; Balenger and Zuk 2015). Second, we asked if movement differed between male morphs in the lab and whether the test substrate (natural grass or artificial foam) matters, allowing us to make a comparison between lab and field and to test for contextual plasticity. We predicted that movement on natural grass would more closely match patterns observed in the wild.

BOX 1 **Cricket CURE at BYU‐Hawaii.**

Course‐based undergraduate research experiences (CUREs) provide high‐impact research opportunities for broader student populations than are usually able to participate in other mentored research arrangements. While CUREs that are integrated into faculty's ongoing research are most effective, this is often difficult for field‐based research that occurs at sites that are geographically distant from their home institutions. To address this challenge, we developed a semester‐long CURE in an animal behavior course that leveraged a two‐institution collaboration model (Smith et al. 2021; see photo of CURE students at the field site in Lā‘ie, HI). Researchers from a “distant” institution, who maintain active field projects, partnered with a “local” institution near their field site, where the CURE was conducted. Undergraduate students involved in the CURE became active contributors to ongoing research and learned about the research process through cooperative, inquiry‐driven experiences. This particular CURE involved multiple levels of mentorship and collaboration, involving PIs (both distant and local), graduate students, undergraduate teaching assistants, and students, resulting in an effective and authentic research experience that generated high‐quality data.

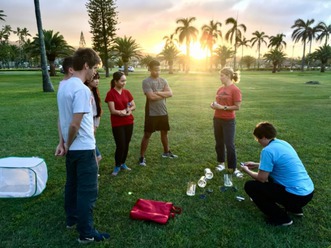

## 2 Methods

### 2.1 Field Experiment

**FIGURE 1:**
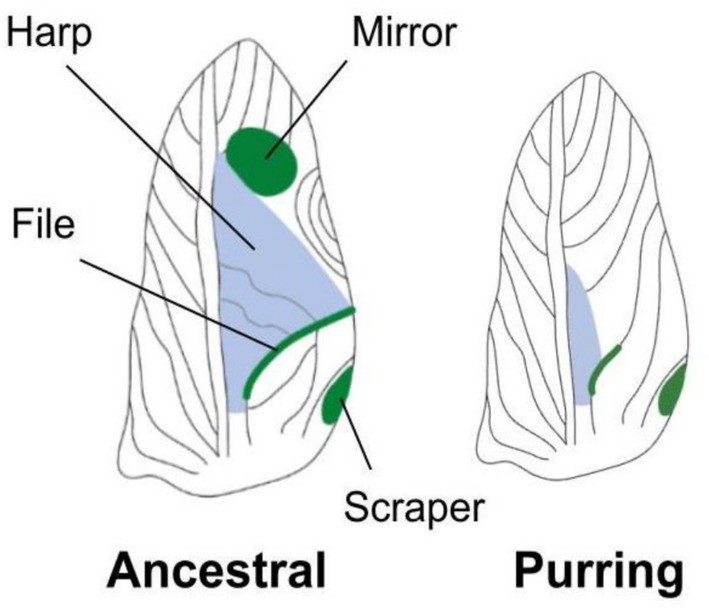
Images of ancestral and purring male wings that differ in major song production structures (labeled), generating differences in song conspicuity. Ancestral males have fully formed, distinctive calling structures on their wings, including the harp, file, mirror, and scraper, while purring males lack most calling structures but do have a reduced file and often a scraper (Figure 1; Bennet‐Clark 2003; Tinghitella et al. 2018). Wings drawn by Gabrielle Welsh.

We conducted a focal‐follow observational study from September to October 2021 on the lawns of the Brigham Young University–Hawaii (BYU‐H) campus in Lā‘ie, Hawaii. This is a long‐studied *T. oceanicus* population that contains both ancestral and novel morphs, namely purring (Figure 1; Tinghitella et al. 2021; Gallagher et al. 2023), as well as a stable population of parasitoid flies (Broder et al. 2022). There are two *T. oceanicus* morphs with similar morphology, purring and silent/flatwing (Zuk et al. 2006; Tinghitella et al. 2018); purring males have a morphology that allows them to produce an audible sound used in mate choice, but silent flatwing males cannot (Tinghitella et al. 2018, 2021). Of the males with attenuated song at this location, 93.2% were purring, and 6.8% were silent in a study of recorded songs conducted 1 year before this study (Gallagher et al. 2023). We hereafter refer to all males with attenuated song as purring, as purring and silent cannot be simply distinguished by eye without recordings. Data were collected by students in BYUH's Animal Behavior course, which was designed as an inter‐institutional CURE (Course‐based Undergraduate Research Experience; following Smith et al. 2021) to conduct research on cricket‐fly interactions on their campus (Box 1).

Prior to beginning the field observations, students gained 3 weeks of training from the local and distant PIs who supervised the work. This training included both focal follow methodology and cricket identification. The PIs and the CURE students first developed an ethogram based on Joern et al. (1986) which classified grasshopper behavior in the wild. The study included behavioral categories of locomotion, courtship activity, feeding, and quiescence (i.e., little visible movement or other activities). Based on our own observations of the crickets, both over years of working with this species and during pilot observational periods, we modified some of the categories we observed by parsing them out into more detailed behavioral categories. For example, “locomotion” was parsed into burrowing (moving under the grass), jumping, and roaming (moving on the surface of the grass), all behaviors that are encompassed by locomotion but are more descriptive in the context of this species. Our ethogram included both state and event categories. Students were only permitted to engage in data collection protocols once they had demonstrated competency and consistency in performing methods according to specifications.

Researchers began field observations at dusk when the nocturnal crickets became active. Students in the course were split into groups of two; one student observed cricket behavior using a focal follow procedure while the other recorded the observations. Students used red headlamps to illuminate the grass; since crickets cannot see this color, this helped to avoid disturbing their natural behavior (Briscoe and Chittka 2001). Researchers conducted continuous focal follows of each male for at least 2 min, and up to 5 min if the cricket remained visible (similar to Joern et al. 1986). The observer walked behind the cricket, maintaining a distance of approximately 0.5 m from the animal. While observers crouched over if necessary, they were never closer than 0.5 m until the end of the observation period when they collected them to identify them (age, sex, and morph). The observer categorized cricket behavior according to an ethogram developed for this project (Table 1) based on the experience of the authors and the literature. When focal follow began, the observer verbally stated the behavior of the focal animal every 15 s or whenever the cricket changed behaviors. For example, an observer would say “R” or “roaming” every 15 s as long as the cricket was walking on top of the grass. If the cricket started moving under the grass, even if this did not fall on a 15‐s mark, they would call out “B” or “burrowing.” Note that we used “B” to indicate movement under the thatch that characterizes this location and not necessarily burrowing under the soil; *T. oceanicus* males do not construct burrows in soil but use and call from shallow cracks in the soil and spaces beneath leaf litter (Zuk et al. 1993, 2001). If a cricket paused a behavior and then resumed, a 5‐s buffer was allowed before relogging it as a new behavior. The recorder kept track of time and recorded data on paper data sheets, which were then input into a spreadsheet.

**TABLE 1:** Ethogram developed for this experiment by students participating in the animal behavior CURE. Note that for this experiment, we were interested in behaviors related to locomotion, such as ST, R, and B.

Code	Description	State or event
B	*Burrowing*: the individual is partially or entirely under the foliage/soil; the individual is oriented with its head down. Event (if the cricket is out of view for greater than 30 s, at which point trial terminates), or State (if the cricket reemerges within 30 s, continuing the trial)	Event/State
J	*Jumping*: cricket pushes itself off the ground and into the air through muscles in its legs	Event
F	*Feeding*: cricket is actively chewing on vegetation; mouth and possibly antennae are actively moving; cricket could be sedentary or moving between foraging sites (within 5 s of each other)	State
M	*Mating*: female mounts male and copulates	State/Event
S	*Stridulation*: male cricket has its wings elevated, either making sound or attempting to make sound, or in between these acts	State
ST	*Stationary*: cricket is in one location without any other active behavior visible (e.g., eating); do not record as ST if the cricket is eating or grooming	State
O	*Oviposition*: the female is actively laying eggs in the substrate. The ovipositor is oriented toward the substrate and is repeatedly touching and lifting off the substrate	State
R	*Roaming*: cricket is actively moving from one location to another, with breaks no longer than 5 s	State
G	*Grooming*: wiping face or antennae with front limbs	State
E	*Encounter*: focal individual comes in physical contact or close proximity (within a body length) to a conspecific	Event
OT	Other	

Once a cricket's focal follow time ended, students picked up the animal to confirm maturity, sex, and male morph and then returned them to their original location. For this study, we only included focal follows of male crickets, which observers classified as either ancestral or purring based on visual inspection of the wings using established differences in morphology. To ensure consistent identification, each member of a research pair examined the crickets and came to a consensus on the morph. Note that the ancestral wing has a distinct mirror that the purring wing lacks, and this can be easily observed on live animals under red or white light (Figure 1). The faculty advisor and an experienced teaching assistant also went from group to group to help supervise the research pairs and ensure proper protocols were followed. If there was ever a question about identification, research pairs would consult with the faculty or teaching assistant before assigning an individual a morph identity. Importantly, observers did not disturb the focal cricket before or during the focal follow period.

To avoid observing the same animal repeatedly, groups moved at least 1 m away before selecting the next focal animal. When groups collected data concurrently, each pair of students had a zone of the large field that did not overlap the zone of other students; pairs could not be within 10 m of each other unless crickets naturally traveled toward another group. These data were collected over 6 weeks and required approximately 360 h of observation.

We calculated the proportion of time that each focal animal spent in each behavioral state by dividing the total time in seconds by the total length of that observation—all of the behaviors of interest for this study are state behaviors. All statistical tests were run in JMP (1989‐2023). We ran three models, one for each of the study's key dependent variables: stationary, burrowing, and roaming. For each, the fixed effect was male type (ancestral male or purring male). For the behaviors stationary (on top of the grass) and burrowing (under the grass), data were not normally distributed and had unequal variance (according to a Levene's test), so we used Median tests (Kasuya 2001). For the behavior of walking (on top of the grass), we log‐transformed the data to meet the assumptions of normality (using visual inspection of QQ plot and Levene's test) and then conducted a t‐test on the log‐transformed data.

### 2.2 Lab Experiment

We used lab‐reared animals from the same focal population as the field experiment for our lab experiment. In August 2023, we collected over 30 adult females from the fields described above. We held the females for at least 5 days under standard husbandry conditions (with moist cotton for water, ad libitum Kaytee rabbit pellets for food, and an egg carton for shelter) during which time the females laid eggs in the moist cotton. We released all crickets back to their natal fields at the end of the 5 days of egg laying. We then transported those eggs back to the lab at the University of Denver. In the lab, the cricket population was maintained in a walk‐in animal husbandry room set to 78° F and on a 12:12 clock‐shifted light: dark cycle (dark from 9 a.m.–9 p.m. and light from 9 p.m.–9 a.m.) with full‐spectrum lights for three generations before testing. The animal husbandry room contained populations of *T. oceanicus* collected from across their range, so all animals could hear ancestral and novel *T. oceanicus* songs during development. Inside the husbandry room, crickets were reared under standard husbandry conditions in plastic containers (42.5 × 30.2 × 17.8 cm for babies and juveniles and 58.4 × 41.3 × 31.4 cm for adults), with moist cotton for water, ad libitum food (Fluker's high calcium cricket diet for babies and Kaytee rabbit pellets for juveniles and adults), and egg carton for shelter.

We tested third‐generation adult males' locomotory behavior in an open‐top glass arena measuring 120 cm × 44 cm. To measure movement, we established a 10 cm × 10 cm grid across the arena to easily score the number of grid lines crossed. To assess contextual plasticity, we used two substrate treatments: artificial foam (a yoga mat) and natural grass. The grass consisted of a hydroponically grown mix of rye, bluegrass, fescue, zoysia, and Bermuda with approximately 2 cm of soil (freshpatch.com). The yoga mat represented an artificial substrate commonly used in similar lab experiments (e.g., Tinghitella et al. 2018, 2021). This was a factorial design with two substrate treatments and two male morphs (*N* = 21 for each treatment/morph combination).

Twenty‐four hours before the experiment, we removed males from the mixed‐sex population boxes and individually housed them in 0.5 L deli cups with ad libitum food, water, and shelter. We gave each a unique ID and visually identified their morph type (again, using established differences in visible wing structures; Figure 1). Note that when these lab trials were run (but not during the field experiment conducted 2 years prior), there was new variation in wing morphology among otherwise ancestral‐looking males (e.g., inclusive of “defiled” from Bailey et al. 2024 and “broken harp” James H Gallager, Personal Communication); this new variation affects structures that cannot be easily observed with the naked eye. So while purring males can be easily visually classified, the ancestral males used in this lab study were likely a mix of both ancestral males and other new morphs that produce signals that differ from ancestral song but are not as attenuated as purring song. We retain the name “ancestral” for this group in this paper but acknowledge the variation. The following day, we measured the width of each male's pronotum (a proxy for body size) and used a random number generator to determine the order in which they received the substrate treatment (artificial or grass).

To initiate the experiment, we placed the test male in the center of the designated arena (artificial or grass) under a 0.5 L deli cup and allowed him to acclimate for 1 min. After 1 min, we lifted the cup, and the cricket was allowed to move throughout the arena for 5 min. During the trial, we recorded the following dependent variables: time until movement (moving a full body length; spinning in a circle did not count), number of grid lines crossed after 3 min, number of grid lines crossed after 5 min, whether they spent more than 75% of their time walking along the wall (yes/no), and if there was an attempt to burrow under the grass or the mat (yes/no). Animals were considered “burrowing” on the artificial substrate if they used their front legs and mandibles to attempt to dig, chew, and pick it apart, exhibiting the same behaviors as when burrowing in the grass substrate. Note that this behavior occurred in the middle of the arena as well as at the edge for both substrates. We recorded the number of lines crossed after 3 and 5 min in case behavior changed over time. We expected there to be no difference between these variables, but animals could have traveled to the edge of the arena in the first 3 min and then remained motionless, for instance. We counted a line as crossed any time an animal moved from one side of the line to the other. If an animal crossed a line and immediately backtracked, crossing that same line again, it was counted both times. However, near the intersection of two lines (at a “corner”) an animal could cross multiple lines simultaneously with less than a single body length; this was only counted as one line if they crossed at the intersection. After the 5‐min observation ended, we placed the cricket back into the deli cup and allowed a 1‐min transition period before testing it in the second substrate treatment. The second trial followed identical methods as described above. After each male completed the two trials, we returned the cricket to an adult mixed‐sex population box in the lab colony. We did not reuse animals. All experiments were completed in August 2024 and were conducted during the animals' scotoperiod within a 6‐h window. The temperature ranged from 26.4°C to 27°C during experiments.

We analyzed the lab data in JMP (version 18). Each model had the same basic structure: a random effect of male ID (since the experiment had a repeated measures design) and two fixed effects: substrate treatment (artificial or grass) and male type (ancestral or purring). The continuous variable “time until movement” was right‐skewed, so we log‐transformed the data to meet the assumptions of an ANOVA (based on visualization of the Q–Q plot and a Levene's Test). We then performed a repeated‐measures ANOVA using the log‐transformed data and the model structure described above (treatment and male type as fixed effects). For the number of grid lines crossed (after 3 and 5 min), we analyzed this count data using GLMMs with a Poisson distribution. For burrowing (yes/no) and whether they walked along the wall, we used GLMMs with a binomial distribution.

## 3 Results

### 3.1 Field Experiment

**FIGURE 2:**
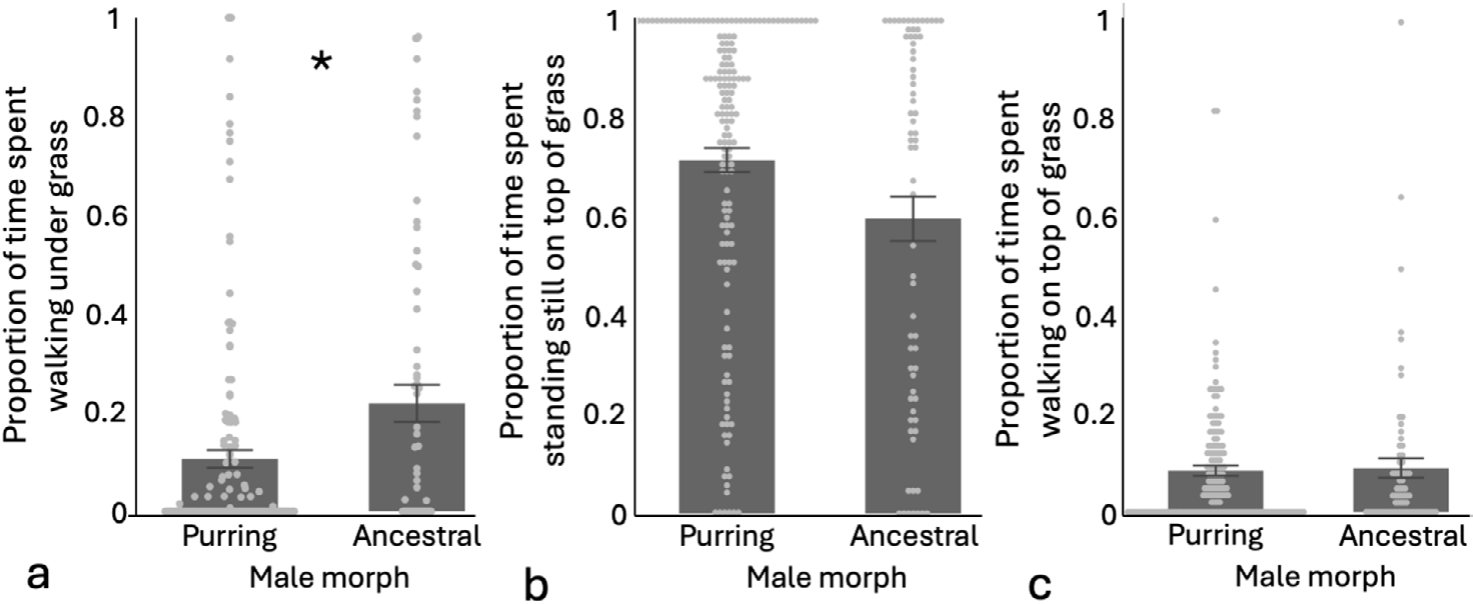
In the field, (a) ancestral males spent more time walking under the grass (asterisk denotes significance). (b) There was no difference in the proportion of time spent standing still on top of the grass or (c) time spent walking on top of the grass between morphs. Shown are means ± standard error, and light gray points represent raw data.

We observed a total of 69 ancestral males and 155 purring males. Ancestral males spent a greater proportion of their time burrowing/moving under the grass than purring males, an average of 22% of their time compared to 11% for purring males (*X* ^2^(1) = 3.82, *p* = 0.05; Figure 2a). There was no difference between morphs in the proportion of time spent standing still on top of the grass; ancestral males spent an average of 60% of their time in this state compared to 72% for purring males (*X* ^2^(1) = 1.69, *p* = 0.19; Figure 2b). There was no difference between morphs in the proportion of time that males spent roaming on the surface of the grass, about 8% of their time for both morphs (*t*_1,224_ = −1.00, *p* = 0.32; Figure 2).

### 3.2 Lab Experiment

**FIGURE 3:**
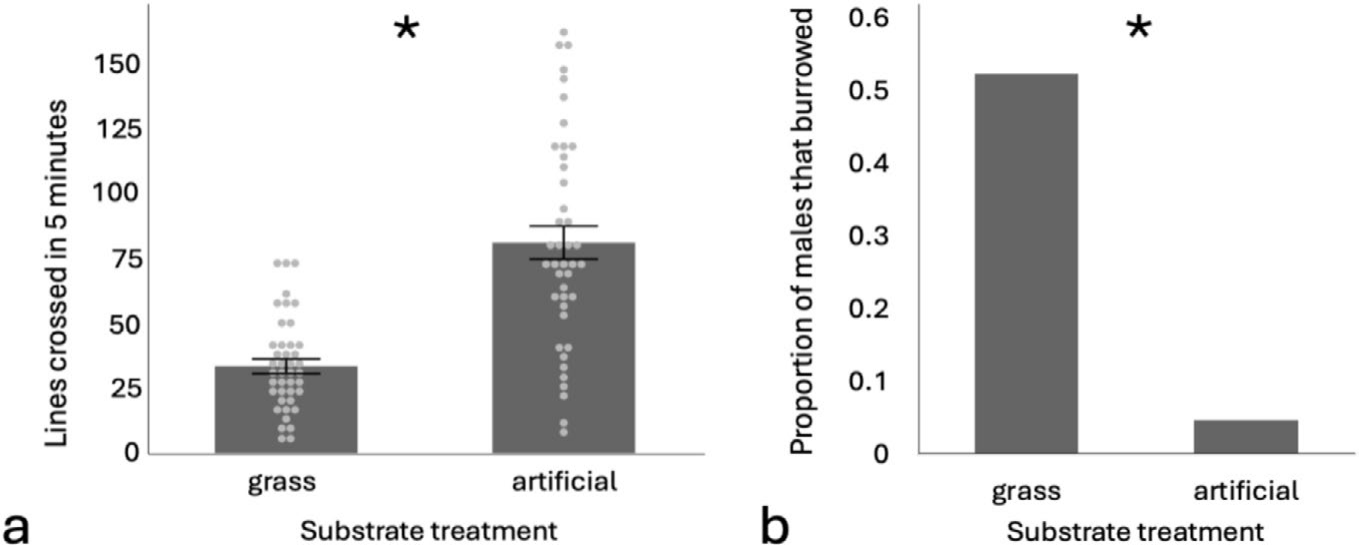
In the lab, behavior differed depending on the substrate. Males were more locomotory on the artificial substrate (a), but burrowed more on the grass substrate (b). Mean ± standard error are shown in a, light gray points represent raw data in a, and the asterisks denote significance.

Crickets did not differ in the latency to movement based on the substrate treatment (*F*_1,41_ = 2.01, *p* = 0.16) or male morph type (*F*_1,41_ = 0.04, *p* = 0.84). Crickets moved more, crossing more gridlines, on the mat compared to the grass after both 3 min (*F*_1,41_ = 448.14, *p* < 0.0001) and 5 min (*F*_1,41_ = 778.16, *p* < 0.0001; Figure 3), but there was no difference between male morphs in lines crossed after 3 min (*F*_1,41_ = 0.62, *p* = 0.44) or 5 min (*F*_1,41_ = 0.38, *p* = 0.54). In 5 min, males, regardless of morph, crossed about 80 grid lines on the artificial substrate compared to only 33 on the natural grass substrate. As expected, the number of lines crossed in 3 and 5 min was highly positively correlated (Pearson's correlation coefficient *r* = 0.957, *p* < 0.0001). Males attempted to “burrow” more in the grass substrate than the mat (*χ* ^2^ = 26.39, *p* < 0.0001; Figure 3), but male morphs were equally likely to burrow (*χ* ^2^ = 0.32, *p* = 0.57). Note that we removed the random effect in this burrowing model because only two individuals burrowed in the mat treatment, making random effects inappropriate as the model fails to converge when a random effect is included. Finally, 75% of the males spent most of their time (75% or more) walking along the wall, and this did not differ by male type (*F*_1,44_ = 0.52, *p* = 0.47) or substrate (*F*_1,81_ = 0.57., *p* = 0.45).

## 4 Discussion

We conducted a lab experiment paired with an in situ focal follow field experiment to observe differences in locomotory behavior of two morphs of the Pacific field cricket: ancestral and novel (purring) under field and lab conditions. The goal was to study how behavior depends on environmental context. In the field, students who were part of a CURE course observed natural locomotory behaviors such as roaming on top of the grass, remaining stationary, and burrowing under the grass. In the lab, male movement was observed in an arena with either an artificial foam or a grass substrate. In the field, ancestral males spent more time walking under the grass (burrowing). This differed from the lab experiment, where both morphs were equally likely to burrow in the grass. We also documented contextual plasticity; both purring and ancestral males moved more on the artificial mat than on the grass after both 3 and 5 minutes.

We set out to test whether there were differences in behavior between the lab and field because such comparisons can help to identify the sources of variation in behavior. Controlled laboratory settings eliminate much of the varied experience that animals have in nature. Comparing the findings in our field‐based focal follows and in lab‐based trials suggests that there are indeed differences in behavior between these contexts. Critically, we found consistent differences in the behavior of the two male morphs in the field, but those differences were not detected in the lab study. Specifically, we found that ancestral males burrowed (moved under the grass) more than purring males in the field, but not in the lab. If burrowing offers some protection from deadly parasitoid flies, we would expect ancestral males to burrow more than purring males since purring males are more protected from flies; flies can detect ancestral songs at 100 m away compared to only 1 m for purring songs (Wikle et al. 2025).

One explanation for the absence of morphological differences in the lab experiment could be that the differences observed in the field are a result of plasticity, so these differences disappeared in the common garden lab experiment. Rearing conditions and adult experience clearly differ in the lab and field. For instance, neither morph experienced parasitism in the lab, and they were reared at higher densities than in the field, which would increase mating opportunities for attenuated morphs that must be in closer proximity to a female to elicit positive phonotaxis (Welsh et al. 2025). In the wild, ancestral males are more attractive to females and parasitoids than morphs with attenuated song (Gallagher et al. 2024). If the morph differences in locomotory behavior we observed in the field are driven by parasitoids, that suggests crickets can respond plastically or learn from experience with flies, which is an exciting area for future study. Alternatively, the absence of morph differences in the lab could be related to the recent and rapid evolution in this population that has produced new variation in the ancestral morph (see methods; Bailey et al. 2024; James H Gallager, Personal Communication). Some males in this population were found to have relatively subtle differences in wing morphology that cause their songs to be anecdotally quieter than pure ancestral males. Thus, the ‘ancestral’ animals in our lab experiment may have represented more than one type. We hypothesize that this new variation may be associated with differences in locomotory behavior (see below). Regardless of the source of the variation we detected in the field, those morph differences were not present in our lab experiment. Thus, our study adds to the growing body of literature espousing the value of field experiments and urging consideration of potential lab effects (Calisi and Bentley 2009).

One surprising finding was that there were no differences in locomotory behavior between morphs in the lab experiment. We expected purring males to move more since some recent laboratory experiments showed that silent flatwing juveniles (Sturiale and Bailey 2023) and adult males (Schneider et al. 2024) moved more than ancestral males. Unlike the silent morph, purring males produce an audible calling song that some proportion of females are attracted to and able to locate (Tinghitella et al. 2018, 2021). Therefore, purring and silent males may differ in locomotion because only purring can produce sound and thus attract mates while stationary. Our results could also relate to the new wing variation that has arisen among ancestral males (described above)—the song produced by these new male types is anecdotally quieter than a true ancestral song. If quieter males all move more than true ancestral males, this may have shifted the behavior of the “ancestral” male group in this study to be more similar to that of purring males who also produce an attenuated song. Future work to fully describe the new wing and song variation we observed and determine its developmental and genetic underpinnings would be valuable.

Indeed, we found contextual plasticity in the lab experiment in that both morphs moved more and burrowed less in the artificial substrate than in the natural grass substrate. Why might this be? It could be easier to walk on the smooth mat than on the grass, so the difference in distance traveled could simply be due to the resistance provided by the substrate. Alternatively, males may have moved more on the artificial substrate because they were more exposed and seeking shelter. However, this is unlikely because regardless of substrate (or morph type), about 75% of males spent the majority of the trial walking along the wall. Finally, there could be contextual plasticity such that the artificial substrate elicits a change in behavior—this increase in movement could be adaptive or maladaptive; developmental plasticity in locomotory behavior has been documented in ancestral and in a silent morph of *T. oceanicus* (Balenger and Zuk 2015). Regarding burrowing, it is clearly easier to burrow in grass than on a foam surface. Even though we observed identical burrowing behaviors on both substrates (digging with front legs and chewing the foam with mandibles), the difference we detected could be an artifact of opportunity. Thus, we do not interpret or extrapolate further.

This research prompted exciting ideas for future work. For example, several additional novel morphs have been described in other locations in Hawaiʻi (e.g., Bailey et al. 2024). Since we observed differences between the novel purring morph and the ancestral morph in the field, it would be interesting to repeat this field experiment in other locations in Hawaii. One challenge in observational field studies is that some characteristics of individuals are unknown. In our study, we have no way of assessing whether a cricket has been parasitized (as this requires holding animals in the lab for more than 1 week to observe pupae emergence). It would be interesting to know if parasitism affects locomotion, and this could be explored in the future in a controlled lab experiment. Additionally, in the lab, a limitation may have been the size and rectangular shape of the arena; three‐fourths of the males in our study spent more than 75% of their time walking along the wall (thigmotaxis). In the future, a larger arena or one with rounded corners or a circular shape may provide a better environment in which to assess movement and behavior. Additionally, on artificial substrates like foam or turf, it is now possible to use video and computer tracking methods to collect much more detailed data on animal movement than was possible in our study (e.g., Schneider et al. 2024; Moschilla et al. 2022). Because we used natural grass that can obscure the animal, we could not use tracking software and used a grid system as a proxy for distance traveled—this has limitations; for instance, an animal that moves in tight circles could earn the same score as an animal that roams over the entire area of the arena. Finally, crickets also communicate using cuticular hydrocarbons (Thomas and Simmons 2009) as well as substrate‐borne vibrations (Broder et al. 2021; Wikle et al. 2023). It could be interesting to consider movement behavior in the context of other communication modalities.

We documented differences between our field and lab studies, namely morph differences in the field that did not appear in the lab. By creating an interinstitutional CURE, we were able to conduct a time‐intensive field experiment that, when paired with a controlled lab experiment, provided a powerful look at movement behavior in this system. We encourage researchers to pair lab experiments with field observations, as this is a robust method for uncovering the importance of experiences animals have in nature for behavioral variation.

## Author Contributions

**Tessa M. Appel:** conceptualization (lead), funding acquisition (equal), investigation (lead), methodology (lead), supervision (equal), writing – original draft (lead), writing – review and editing (equal). **Sterling Kerr:** data curation (equal), formal analysis (supporting), investigation (lead), methodology (equal), supervision (equal), writing – original draft (equal), writing – review and editing (equal). **Robin M. Tinghitella:** conceptualization (lead), funding acquisition (equal), methodology (equal), project administration (equal), supervision (equal), writing – original draft (equal), writing – review and editing (equal). **Spencer Ingley:** conceptualization (equal), data curation (equal), formal analysis (supporting), investigation (equal), methodology (equal), supervision (lead), writing – review and editing (equal). **Gabe Meyer:** data curation (equal), investigation (equal), writing – original draft (equal), writing – review and editing (equal). **E. Dale Broder:** conceptualization (lead), data curation (equal), formal analysis (lead), funding acquisition (equal), investigation (supporting), methodology (equal), project administration (lead), visualization (lead), writing – original draft (lead), writing – review and editing (equal).

## Funding

This work was supported by the Directorate for Biological Sciences, University of Denver's Undergraduate Research Center Summer Research Grant to T.A. and NSF grants to R.M.T. and E.D.B. (IOS 2240950 and IOS 1846520).

## Conflicts of Interest

The authors declare no conflicts of interest.

## Supporting information


**Data S1:** ece372804‐sup‐0001‐DataS1.xlsx.

## Data Availability

Data are available as a supplement.
